# Utility of the AngelMed Guardian System in myocardial bridging: a case report

**DOI:** 10.1186/s43044-024-00573-4

**Published:** 2024-10-10

**Authors:** Lakshmi Rao, Arjun Chadha, Anila Rao, Anurag Khandavalli, Surya Rao

**Affiliations:** 1 McLaren Macomb Medical Center, 1000 Harrington St, Mount Clemens, MI 48043 USA; 2 AdventHealth Cardiovascular Institute, Port Orange, FL USA

**Keywords:** Myocardial bridging, AngelMed Guardian System, Guardian device

## Abstract

**Background:**

Myocardial bridging (MB) is a known congenital anomaly in which a segment of the coronary artery transverses from the epicardium through the myocardium. MB may clinically manifest as recurrent angina, acute coronary syndrome, ventricular dysrhythmia, and even sudden cardiac death. On electrocardiogram (EKG), MB can present with findings consistent with significant ST-segment changes. The AngelMed Guardian System (the Guardian device) was developed in an effort to optimize the time from the onset of myocardial ischemia to intervention. The device analyzes myocardial electrical changes and alerts the patient to seek emergent medical evaluation if an acute ST-segment deviation is detected. We describe the first documented case of the Guardian device detecting acute ST-segment changes secondary to myocardial bridging.

**Case Presentation:**

A 50-year-old male, with a history of percutaneous coronary intervention of the proximal left anterior descending (LAD) artery, presented to his cardiologist’s office with reports of recurrent, atypical chest pain. The Guardian device was implanted. One month after implantation, the patient experienced chest pain and was alerted by the Guardian device to seek emergent medical evaluation. Initial EKG and high-sensitivity troponins were negative for acute ischemia. Guardian device interrogation revealed significant ST depressions, encouraging further ischemic evaluation. The exercise myocardial perfusion imaging revealed an apical reversible defect. Left heart catheterization revealed a large segment of mid-LAD MB with the patient’s previous LAD stent noted to be widely patent along with no evidence of new obstructive coronary disease. Following the identification of the MB, medication regimen adjusted and the patient denied recurrence of chest pain or Guardian alerts.

**Conclusions:**

We describe the first documented case of the Guardian device detecting acute ST-segment changes that were secondary to myocardial bridging. The Guardian device appropriately documented an ischemic event not appreciated with initial EKG or troponin testing. With the assistance of the Guardian device, we were able to rapidly identify MB as the cause for the ischemic EKG changes and subsequent abnormal stress test, optimize medical management, and prevent recurrent atypical chest pain along with office and hospital visits.

## Background

Myocardial bridging (MB) is a congenital anomaly characterized by a segment of the epicardial coronary artery that transverses through the myocardium [1]. Autopsy results reveal this congenital anomaly to be present in as many as a third of adults and are most commonly present in the left anterior descending (LAD) artery [1]. Clinically, MB may present as recurrent angina, acute coronary syndrome, ventricular dysrhythmia, and even sudden cardiac death [1]. Resting EKG can range in patients with MB from normal to ST-segment abnormalities [1].

It is known that the duration of myocardial ischemia is directly correlated with irreversible cardiomyocyte injury, with an ischemic time greater than 20 min being associated with cardiomyocyte death. The AngelMed Guardian System (the Guardian device) was developed in an effort to optimize the time from myocardial ischemia onset to hospital presentation (door time) and ultimately to time of coronary intervention and/or restoration of TIMI-III flow (balloon time) [3]. The Guardian device is composed of 2 main components: an implantable medical device with a right ventricular lead and an external monitoring device [3]. The Guardian device is programmed with an algorithm that triggers an emergency alert whenever there is a ST-segment shift of more than 3 standard deviations from a patient’s baseline electrogram [3].

We describe the first documented case of the Guardian device detecting acute ST-segment changes secondary to myocardial bridging.

## Case Presentation

A 50-year-old male, with a history of a proximal LAD stent 6 months prior after an acute coronary syndrome (ACS) event, presented to his cardiologist’s office with recurrent, atypical chest pain. Given his recurrent chest pain, elevated risk for future ACS events, and recurrent negative electrocardiograms (EKGs) for ischemia, a Guardian device was implanted for further monitoring.

One month after implantation of the Guardian device, the patient began to have a dull left-sided chest pain while sitting and watching television. The Guardian device vibrated in his chest and an emergency alert on his handheld device was activated. He immediately presented to the emergency department for medical evaluation. EKG and high-sensitivity troponins during his evaluation were negative for acute ischemia. Interrogation of the Guardian device revealed significant ST depressions on the intracardiac electrogram. Given the alerts from the Guardian device and clinical symptoms, an exercise myocardial perfusion imaging (MPI) test was performed. The exercise MPI revealed a small in size and moderate in intensity apical reversible defect. Notably, he also experienced chest pain with exercise during the examination. Given the positive stress test and symptomatology, he underwent a left heart catheterization (LHC). LHC revealed a large segment of mid-LAD myocardial bridging and a patent LAD stent. No significant angiographic stenosis was noted in the right coronary artery or left circumflex artery. ST-segment changes noted by the Guardian device were noted to be from the significant LAD myocardial bridging and his metoprolol tartrate was increased from 25 mg twice daily to 75 mg twice daily. After the medication adjustment, the patient has not had recurrence of his atypical chest pain, and no further Guardian alerts have been found.

## Conclusions

Although myocardial bridging (MB) is generally asymptomatic, it has been associated with clinically relevant ischemic symptoms [1]. Furthermore, it has been associated with increased risks of major adverse cardiac events and myocardial ischemia [2]. During the systolic phase of the cardiac cycle, the surrounding myocardium contracts, leading to compression of the tunneled coronary artery segment [1]. Systolic compression of the coronary artery yields a delay in the early diastolic hyperemia (most prominent in the subendocardial tissue). This dynamic interplay is manipulated by several factors, including sympathetic tone and coronary steal by collateral branches.

The AngelMed Guardian System (the Guardian device) has two components: an implantable medical device (IMD) and external device (EXD) [3]. IMD is subcutaneously placed in the anterior chest and senses myocardial electrical changes from a lead that is actively fixated in the right ventricular apex. Every 90 s, the device analyzes a 10 s electrogram. The device compares the ST-segment deviation relative to the preceding PQ interval to a 24 h personalized baseline ST-segment electrogram [3]. If an acute ST shift is detected, the IMD vibrates and EXD beeps and flashes with colored indicators. This alerts the patient to seek medical evaluation.

The Implantable Cardiac Alert System for Early Recognition of ST-segment elevation myocardial infarction (ALERTS trial) is a phase III multicenter, randomized trial conducted to assess the clinical impact of the Guardian System and expand on the findings of the Cardiosaver and DETECT phase I clinical studies [4]. The Guardian System, utilizing a noise reducing dual-baseline ECG analysis, has been shown to significantly decrease the number of subjects impacted with a cardiac or unexplained death, new Q wave, or detection to presentation time > 2 h for a documented coronary occlusion event (6.5% vs. 3.1%; posterior probability [*R*_*t*_ < *R*_*c*_] = 0.991). Furthermore, the Guardian System also decreased the time of ST-segment deviation to arrival time at a medical facility (51 min vs. 30.6 h; posterior probability [patient < control group] > 0.999). In the ALERTS trial, several alarms triggered for atrial fibrillation, tachycardia, bradycardia, new-onset bundle branch block, transient heart block, anemia, and hypokalemia. There are no documented alarms being triggered from myocardial bridging.

In regard to our patient, myocardial bridging resulted in intermittent, clinically significant ischemia. These episodes were appropriately sensed by the Guardian device and were documented as ST depressions. Given that these ischemic episodes were sensed by the Guardian device and not by troponin or EKG, our case suggests that this device may assist in increasing the specificity of the overall diagnostic testing for ischemia. Proper identification of the MB causing the ST depressions allowed for appropriate titration of our patient’s beta-blocker therapy to allow for increased diastolic filling time, decreased peak heart rate, and decreased contractility and compression of the MB segment. The increase in beta-blocker therapy in our patient prevented further hospital visits and episodes of chest pain. Our case showcases the Guardian device’s potential to identify and subsequently manage clinically significant MB.

## Data Availability

Not applicable.

## Abbreviations

MB: Myocardial bridging
EKG: Electrocardiogram
The Guardian device: AngelMed Guardian System
LAD: Left anterior descending
ACS: Acute coronary syndrome
MPI: Myocardial perfusion imaging
LHC: Left heart catheterization
IMD: Implantable medical device
EXD: External device
